# How do I remove my clinical glasses? A qualitative study with mental healthcare professionals about patient experience film

**DOI:** 10.1177/10398562261425554

**Published:** 2026-02-13

**Authors:** Elisabeth A. Hoekman, Barbara C. Groot-Sluijsmans

**Affiliations:** Faculty of Science, Health Sciences, 1190 Vrije Universiteit, Amsterdam, The Netherlands

**Keywords:** psychiatric professional perspectives, patient experience, person-centered care, training and education, qualitative research

## Abstract

**Objectives:**

Globally, mental health disorders represent a significant public health challenge. There is growing interest in patient-centered care within psychiatric practice, emphasizing the need to understand patients’ lived experiences. Films illustrating patient perspectives have been proposed as a means to foster empathy among mental health professionals.

**Methods:**

This qualitative study investigates how mental health professionals experience patient experience films.

**Data Collection and Analysis:**

This study uses reflexive thematic analysis of interviews and focus groups.. with 23 Dutch healthcare professionals (psychiatrists, nurses, general practitioners, and medical interns).

**Results:**

This study shows: (1) Patient-experience Films Aren’t on Our Radar, (2) Our take on the film: from moved to mystified, (3) We See Big Value Here—Especially for Those Just Starting Out, and (4) Making It Work: Ideas and Obstacles for Using Films in Training. Patient-experience films are seen as potentially valuable for trainees, but institutional resistance presented significant barriers to integration.

**Conclusion:**

Systemic and structural modifications are recommended to optimize film efficacy as training tools.

## Introduction

Globally, mental health disorders are a significant public health concern, with approximately one in eight individuals affected.^1^ In the Netherlands, the prevalence has risen notably; in 2021, 18% of young people aged 12 to 24 reported being mentally unhealthy, up from 11% in 2019.^2^ Furthermore, based on the most recent data from the Netherlands Mental Health Survey and Incidence Study 3 (NEMESIS-3), conducted between 2019 and 2022, approximately 48% of Dutch adults aged 18 to 75 have experienced at least one mental disorder in their lifetime. This includes mood disorders, anxiety disorders, and substance use disorders.^3^ In 2021, 515,369 patients received specialized mental health treatment.^4^

Person-centered care (PCC) has gained global attention, emphasizing individualized healthcare that respects patients’ needs and values. The WHO Patient Safety Action Plan 2021–2030 incorporates PCC principles.^5^ PCC involves treating individuals with dignity and providing coordinated, personalized care.^6^ Despite its benefits, implementing PCC faces challenges, as clinical practices often prioritize medical conditions, financial constraints, or procedural efficiency over personal patient needs.^7^ This misalignment can lead to patients feeling disconnected from their caregivers, resulting in dissatisfaction.^8^ Such disconnects are evident in various patient narratives, including blogs, news articles, and medical journals, where individuals express a desire for more empathy, respect, and personalized attention during their care.^9–11^

Research indicates that PCC can significantly enhance patient outcomes.^12^ Effective crisis care involves not only addressing immediate medical needs but also fostering meaningful connections, building trust, and ensuring patients feel heard and understood. This necessitates a shift from a disease-focused approach to one that recognizes patients as individuals with unique preferences Ref. 13. One method to contribute to this shift is through patient-experience films, which depict real-life healthcare interactions, highlighting patients’ emotional, physical, and social journeys.^14^ These films can be produced by patients but can also come in genre type like a documentary, short films etc.

Despite some early interest, the academic exploration of patient-experience film as a tool in psychiatric training remains limited, with most studies now over a decade old and potentially lacking relevance to current educational contexts.^15^ Patient-experience films provide insights into the lived experiences of individuals with mental health disorders, thereby enhancing empathy and comprehension. Studies have demonstrated their effectiveness as valuable learning resources.^16–18^ Using patient-experience films in education has been increasingly integrated into medical curricula to elucidate the complexities of mental disorders.^16,17^ Although recent literature on this topic is limited,^18^ report that using patient-experience film in mental health education through weekly workshops enhances empathy and active listening skills among students, positively influencing their clinical perspectives. However, there is limited research on the impact of patient-experience films on seasoned healthcare professionals, particularly those in psychiatry.

Previous studies have primarily focused on utilizing patient-experience films to educate medical students, aiming to foster empathy and improve understanding of patient perspectives.^18,19^ Few have explored how such films are perceived by healthcare professionals, especially those directly involved in psychiatric care. Addressing this gap, the current study investigates how psychiatric healthcare professionals experience patient-experience films. The research question posed is: “How do healthcare professionals in psychiatry experience a patient-experience film?” Findings from this study may inform subtle yet meaningful improvements in psychiatric care practices.

## Method

This research adopted a qualitative, exploratory design grounded in a social constructivist approach,^20^ (p. 66), recognizing that experiences are socially and culturally constructed within the specific context of the Netherlands in this study. This study centered around the patient-experience film “Zielsverduistering,” created by Sem de Boer and Stijn Pruijsen (see Figure 1). The film depicted Sem’s personal experiences with psychiatric institutions during her anorexia treatment, illustrating the broader impact on her family. This film was chosen as Sem wanted to use her artistic film as an educational film for mental health staff, which led to the research question and study. So Sem was the initiator of this study, shared her film and network with us, and will utilize the results of this study.

**Figure 1.**
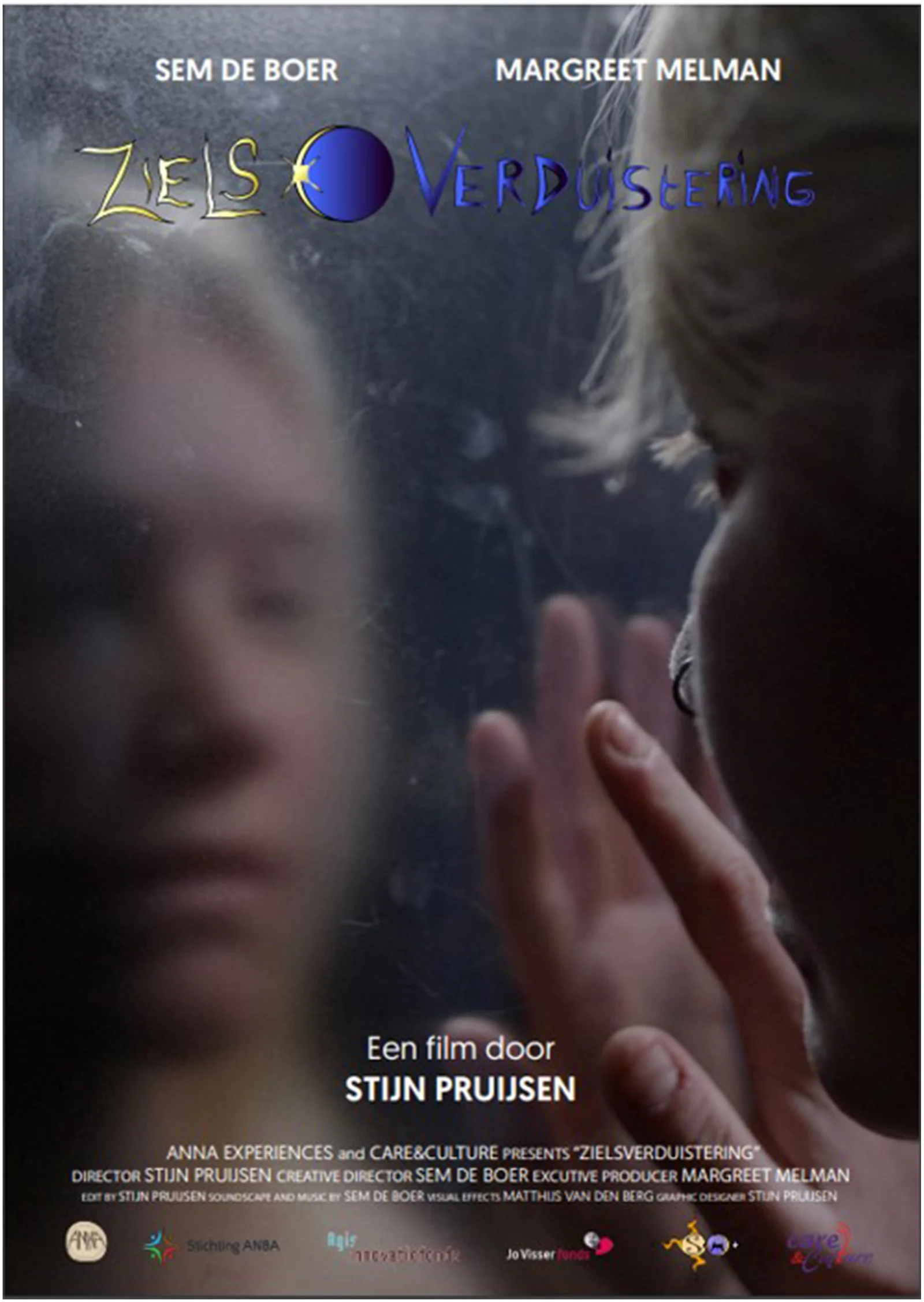
The film poster of the documentary “Soul Eclipse” (in Dutch: “Zielsverduistering”).

We used Sems film as a “case” in our case study to learn from it and its context as an illustrative example. We conducted interviews with people who attended the film’s premiere to gain insights into their individual experiences. Additionally, we conducted focus groups with people, in which we took time to watch the patient-experience film as a starting point, to gain insight into experiences from a group of people with no personal connection to the film’s makers (see Figure 2).

**Figure 2.**
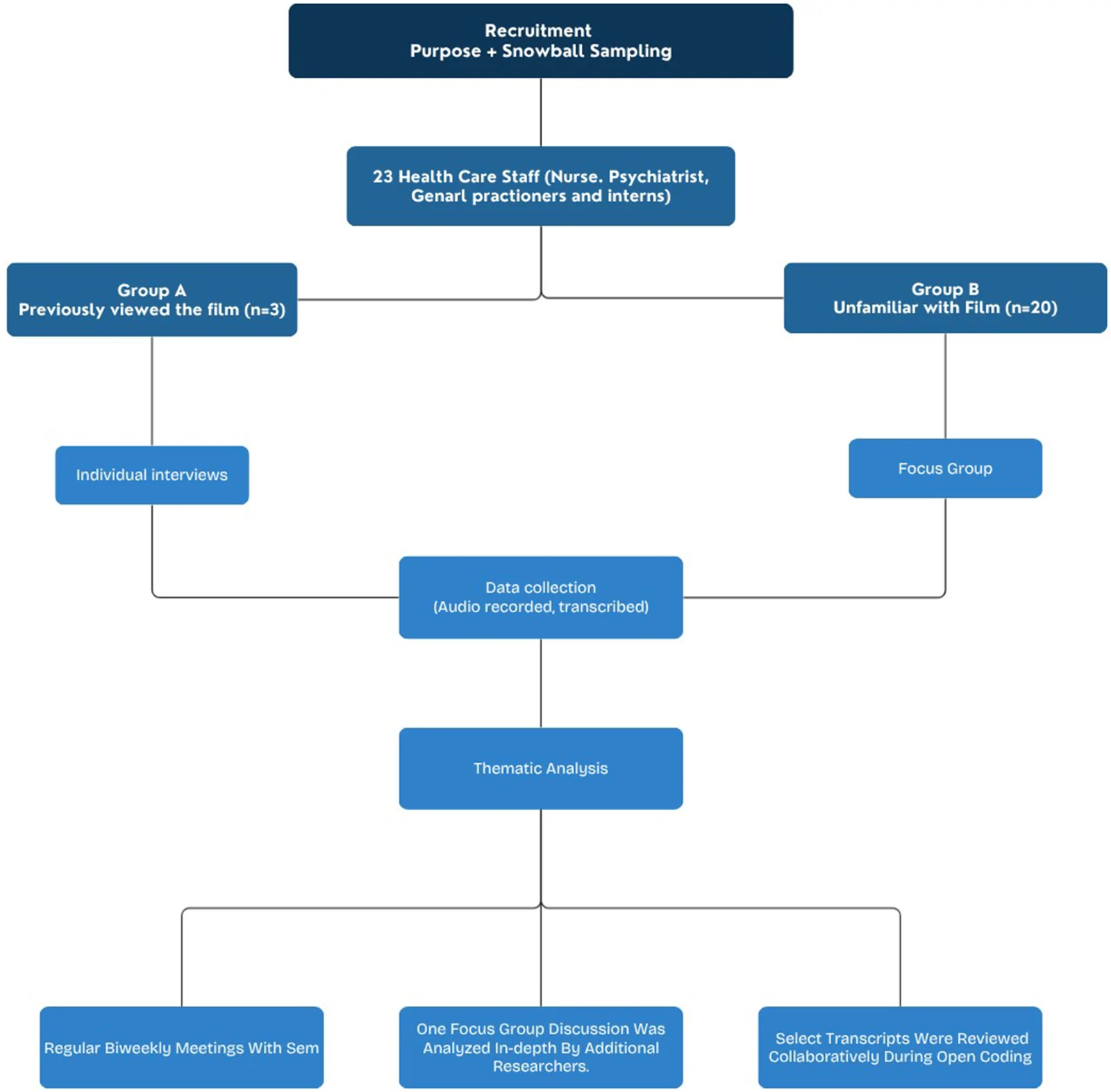
Flowchart recruitment and analysis.

Participants included 23 health staff, including professionals working in psychiatric care in the Netherlands, comprising psychiatrists, psychiatric trainees, nurses, nurse specialists, general practitioners in training, and interns. Recruitment utilized purposive sampling to ensure diverse representation,^20^ (p. 160), supplemented by snowball sampling to expand the participant pool,^20^ (p. 165). Participants were divided into two groups: those who had previously viewed the film and those who had not. Individuals who had previously watched the film participated in individual interviews. These participants also knew the patient and/or had partaken in the film. At the same time, those unfamiliar with the film attended focus groups where the film was screened. Most participants were recruited via existing networks, including people who attended the film’s premiere, Sem’s and her mother’s networks, and the researcher’s mother, who facilitated further recruitment.

Separate interview guides (see Appendix) were created for individual interviews and focus groups, addressing emotional and cognitive responses to the film, its perceived relevance to clinical practice, and potential behavioral changes. The focus group guide included interactive elements to stimulate group discussion, whereas the individual interview guide emphasized reflective questions,^20^ (p. 195). Interviews lasted 45–60 min and were conducted face-to-face or via video conferencing, based on participant availability. Focus groups ranged from 1.5 to 2 h and occurred face-to-face or in hybrid formats. All sessions were audio-recorded and transcribed verbatim. In line with reflexive thematic analysis, data adequacy was judged through analytic sufficiency, the point at which further data collection no longer substantially deepened or diversified the developing interpretations.^21^ The final sample of 23 participants provided sufficient richness, depth, and diversity to address the study aims. Thematic analysis followed an inductive coding process of all data (interviews and focus groups), performed primarily by one researcher. The analysis involved open coding to identify initial concepts, axial coding to group related codes, and selective coding to refine overarching themes,^20^ (p. 467). To enhance analytical rigor, select transcripts were reviewed collaboratively during open coding, and one focus group discussion was analyzed in-depth by additional researchers. Regular biweekly meetings with Sem provided further insights and iterative refinement.

Ethical approval was granted by the Research Ethics Review Committee of the Faculty of Science (BETCHIE). Participants provided verbal or digital informed consent, ensuring voluntary participation and data anonymity, with freedom to withdraw at any time. Data were securely stored and deleted upon study completion. Additionally, a research diary and logbook were maintained for reflexive documentation. Given the single researcher design, it was essential to reflect on positionality, acknowledging the researcher’s educational and personal relationships to certain participants. Measures to mitigate potential biases included external transcript reviews and ongoing reflexivity to acknowledge undue influence from personal connections.

## Results

It is notable that the majority of the participants work in hospital settings and have a wide range of experience in healthcare, from less than a year to over 45 years (Table 1). The sample predominantly consists of psychiatrists (*n* = 4) and psychiatrists in training (*n* = 6), alongside nurses (*n* = 3), general practitioners (*n* = 1), general practitioners in training (*n* = 2), interns (*n* = 3), and a nurse specialist (*n* = 1) (Table 1). This diverse group offers a comprehensive perspective, reflecting various professional backgrounds and levels of expertise within the healthcare sector.

**Table 1.** Overview of characteristics of the participants.

Respondent	Type of health care worker	Time in healthcare	Work location	Interview/focus group
R1	Nurse	17 yrs+	Hospital	Interview
R2	Nurse	25 yrs	Ambivalent care	Interview
R3	Nurse	45 yrs	Retired	Interview
R4	Psychiatrist	20 yrs	Hospital	Focus group (1)
R5	Psychiatrist	28 yrs	Private practice	Focus group (1)
R6	Psychiatrist	25 yrs	Private practice	Focus group (1)
R7	General practitioner in training	10 yrs	Hospital	Focus group (2)
R8	Doctor unspecified	10 yrs	Hospital	Focus group (2)
R9	General practitioner in training	12 yrs	Hospital	Focus group (2)
R10	Psychiatrist in training	5 yrs	Hospital	Focus group (2)
R11	Intern	9 mths	Hospital	Focus group (2)
R12	Intern	5th year (medicine)	Hospital	Focus group (2)
R13	Intern	5th year (medicine)	Hospital	Focus group (2)
R14	Psychiatrist in training	2 yrs	Hospital	Focus group (2)
R15	Senior intern	6th year (medicine)	Hospital	Focus group 2)
R16	Psychiatrist	35 yrs (25 yrs as a psychiatrist)	Hospital	Focus group (2)
R17	Psychiatrist in training	6 yrs	Hospital	Focus group (2)
R18	Psychiatrist in training	5 yrs	Hospital	Focus group (2)
R19	General practitioner	7 yrs	Hospital	Focus group (2)
R20	Psychiatrist in training	8 yrs	Hospital	Focus group (2)
R21	Psychiatrist in training	6 yrs	Hospital	Focus group (2)
R22	General practitioner	5 yrs	Hospital	Focus group (2)
R23	Nurse specialist	30 yrs	Hospital	Focus group (2)

Analysis of the interviews and focus groups revealed four main themes: (1) Patient Experience Films Aren’t on Our Radar, (2) Our take on the film: from moved to mystified, (3) We See Big Value Here—Especially for Those Just Starting Out, and (4) Making It Work: Ideas and Obstacles for Using Films in Training. Each theme contained several subthemes that provided deeper insight into healthcare workers’ perspectives on patient perspective films (see Figure 3).

**Figure 3.**
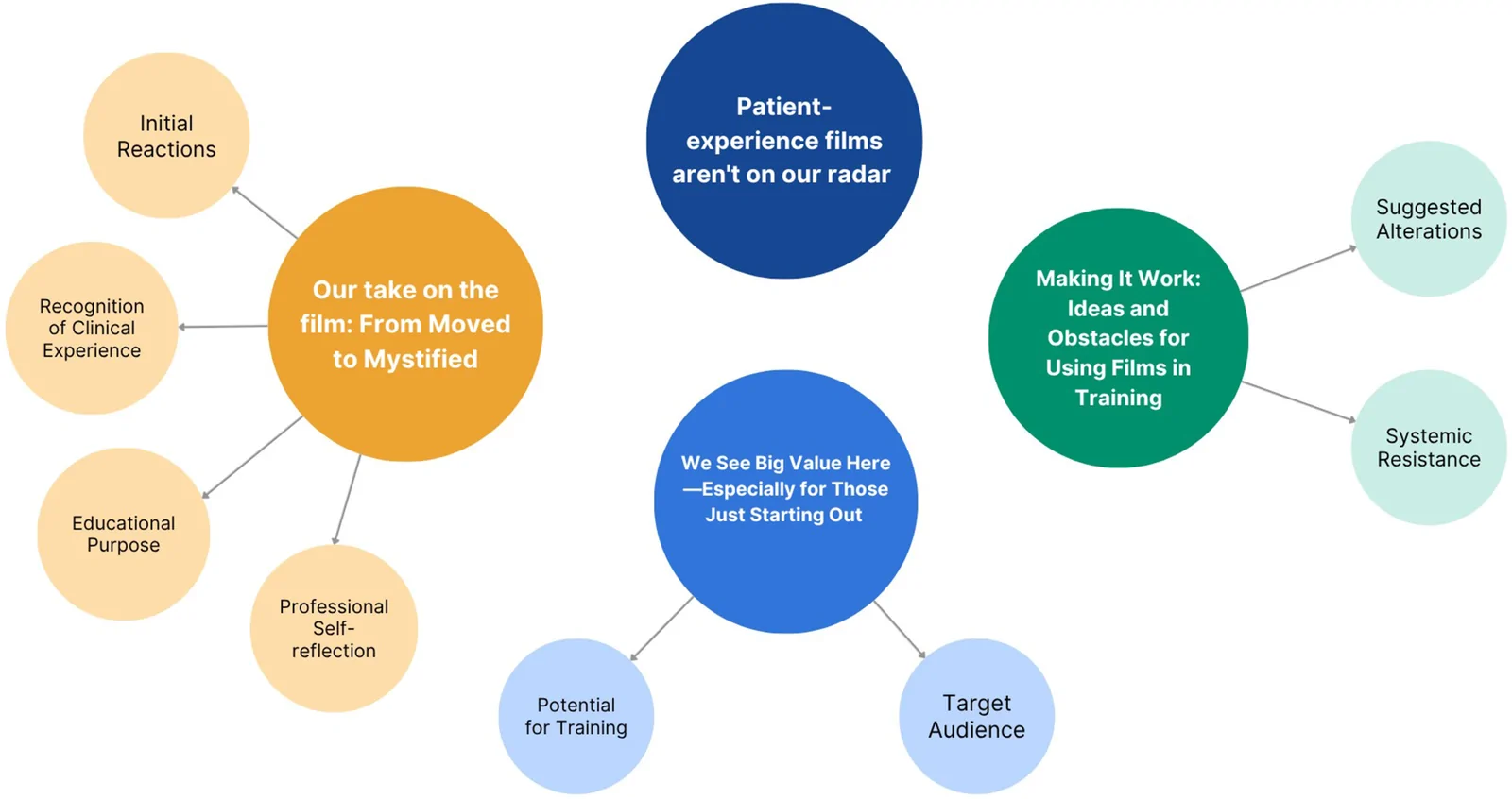
Themes from the analysis.

### Patient-experience films aren’t on our radar

Healthcare professionals rarely engaged with patient perspective films, typically preferring written patient narratives or direct patient interactions. Those who had encountered patient-experience films generally did so either during their medical education or due to personal connections with patients involved in creating such films. Participants often perceived films as peripheral rather than integral educational tools.

“*I really do read books about people, about their psychiatric history. I also have patients who publish their own poetry collections or write down a biography… but films aren’t something I’ve engaged with regularly*.” (R5, Psychiatrist, 28 yrs experience)

### Our take on the film: From moved to mystified

#### Initial reactions

Initial reactions varied significantly among participants. Some professionals found profound emotional connections with the patient’s vulnerability and courage depicted in the film. Conversely, others experienced discomfort or confusion due to the film’s artistic elements or the unclear intent behind its production.

“*I found her insight about that insecurity, I thought it was brave. And that conversation by the wood-burning stove, I also found brave… She beautifully describes her vulnerability. And you actually see that. She is really a very vulnerable woman.*” (R4, Psychiatrist, 20 yrs experience)

However, artistic choices in the film were polarizing. Some participants found them distracting, stating:

“*I think in this film, everything around it*—*the artistic aspect plays a very big role. That’s how it feels to me. And because of that, I occasionally disengaged. Whereas I thought about the content, oh no, That’s what it’s really about that I found interesting*.” (R23, Nurse Specialist, 30 yrs of experience)

#### Recognition of clinical experience

Participants consistently recognized familiar clinical themes presented in the film, particularly regarding patient autonomy, logic underlying the illness, and resistance to treatment. Professionals underscored the complexities of treating patients who are deeply invested in maintaining control as part of their coping mechanism.

“*It is very important… that people find that hook at some point, that they get a bit of an idea of why they want to hold on to it so tightly. That she can already explain that we grew up in very turbulent times. This is how I find my grip.*” (better translated) (R6, Psychiatrist, 25 yrs experience)

Participants also highlighted the accurate depiction of family involvement, underscoring the variability in family dynamics and their impact on treatment outcomes.

#### Educational purpose

For experienced professionals, the film primarily reinforced rather than introduced new insights, reiterating known clinical challenges. Participants felt the primary educational value lay in its potential to remind healthcare providers about the importance of individualized care and considering the patient’s personal background.

“*I especially liked how you not only see the patient and the hospital—the whole story around it—and that you actually have to make care more individualized to the patient*.” (R11, Intern, 9 months experience)

Several noted the film’s ability to vividly illustrate theoretical concepts such as the systemic nature of illness and recovery, enhancing their appreciation of patient experiences.

#### Professional self-reflection

The film prompted substantial reflection on participants’ clinical practice and professional attitudes. Discussions around the unclear purpose of the film encouraged self-awareness among participants about their expectations and reactions as healthcare professionals.

“*Maybe that says more about us as doctors, that we really want to know what the assignment is, what we’re looking at, what the goal is*.” (Participant, Focus Group 2)

### We see big value here—especially for those just starting out

#### Potential for training

Participants generally recognized patient-experience films as valuable educational resources. Films were seen as beneficial in maintaining empathy and clinical adaptability, particularly useful as refresher tools or in addressing contemporary changes in medical practice. Professionals suggested that films, when structured effectively, could significantly enhance clinical empathy and understanding, offering richer insights than traditional educational materials.

“*I think it can also be positive in terms of how we reflect on our own actions and behaviour. However, if that was really the aim, I think I may have missed it a little in this film, which tells her own story, which is very beautiful. But I think it would also be nice if we could reflect on what we as carers can do to harm people, but also what we can do to help them.*” (R8, General practioner in training, 10 yrs experience)

“*I think that per illness you have to think very carefully about how you portray it, and with what exact purpose. Or an interesting question from a larger group who wants to retrain. Because I wouldn’t be waiting for a training with just a number of stories that are all depicted. If you really want to make a training out of it, I think you have to make that adjustment of the film in collaboration, with just such a session as now. Or from the patient—what she missed with the caregivers, what she wants to teach us. That is very important, I think*.” (R19, General practitioner, 7 yrs of experience)

#### Target audience: Early-careers

While views varied, there was a consensus that early-career professionals, including interns and residents, would particularly benefit from these films. However, participants also advocated broader usage across hospital departments dealing with patients experiencing similar mental health challenges, arguing for universal relevance within healthcare environments.

“*You meet them in the ICU; you meet them in the children’s department. Those are the places where you encounter them. But yes, you can also get them in surgery… So this is relevant for an entire hospital*.” (R3, Nurse, 45 yrs experience)

“*I was thinking, maybe during my internship or something, I think I would have really liked it. You often had no idea about anything. You sit in the lecture hall and they bombard you with all these diseases and you think, yeah, fine. But actually, you only remember them once you’ve seen someone with them. So in that respect, I would have enjoyed it. I did enjoy it now, though…*” (R7, General practitioner in training, 10 yrs experience)

### Making it work: Ideas and obstacles for using films in training

#### Suggested alterations

Participants emphasized specific modifications to increase films’ educational effectiveness. Common suggestions included reducing artistic and abstract elements, shortening the length for improved accessibility, and integrating structured panel discussions to deepen professional engagement and understanding.

“*Then you should also add a kind of panel so that you get a discussion*.” (R1, Nurse, 17 yrs+ experience)

“*I found the ending rather long-winded. It sometimes made me feel a bit impatient*.” (R5 Psychiatrist, 25 yrs experience).

#### Systemic resistance

Institutional resistance by health professionals emerged as a significant barrier, characterized by skepticism about the educational validity of films portraying patient experiences. Participants described encountering pushback rooted in perceived inadequacies regarding direct clinical training value, indicating systemic hesitancy to adopt unconventional educational methods.

“*Because they don’t find it educational for professionals, because it’s too much an experience story. That rejection of the hospital to, let’s say, we don’t want to advertise*.” (R3, Nurse, 45 yrs experience)

This resistance underscores broader organizational reluctance, suggesting the need for advocacy and strategic adjustments in the integration of such tools into professional education frameworks.

## Discussion

This study contributes to filling a notable gap in the literature by exploring the impact of patient-experience films on healthcare professionals within psychiatric settings. Four central themes emerged: limited engagement with patient perspective films despite recognized potential; diverse emotional and clinical reactions; educational value primarily identified for early-career professionals; and significant systemic barriers to integration.

Healthcare professionals in this study predominantly preferred written patient narratives or direct interactions over patient perspective films, aligning with findings by Ogston-Tuck and colleagues.^17^ This preference highlights professionals’ familiarity with traditional forms of learning and suggests a missed opportunity to leverage visual narratives in enhancing emotional engagement and empathy.^15^ Our findings indicate professionals often default to clinical detachment, limiting the emotional connection essential for understanding patient experiences deeply. Such detachment aligns with professional expectations but may hinder fully appreciating the subjective dimensions of patient narratives.^17^

Participants recognized key clinical elements such as patient autonomy, logic behind illness, and treatment resistance effectively portrayed in the film. This resonates with previous work by Adams and colleagues,^14^ demonstrating the potential of visual narratives in complementing traditional educational methods. Particularly for early-career professionals, patient perspective films could enhance clinical training by illustrating theoretical knowledge in a vivid, emotionally engaging format. The systemic challenges identified, including institutional skepticism toward the educational validity of such films, echo prior literature documenting resistance to integrating non-traditional educational tools in healthcare environments.^14,22^

While films offer distinctive emotional and immersive experiences that foster empathy and deepen understanding,^15^ our study reveals diminishing emotional engagement among experienced professionals who prioritize clinical utility. This observation emphasizes the importance of tailored educational strategies that address varying professional development stages. Films could therefore benefit from adaptations like structured discussions or panel sessions to clarify their educational objectives and increase practical relevance.

Several strengths underpin the contribution of this research. The diverse participant group, encompassing various professional roles and experience levels, provided rich insights into professional perspectives within psychiatric care. The combination of interviews and focus groups allowed exploration of both individual reflections and group interactions. The inductive methodology facilitated the emergence of unanticipated themes, such as tensions between clinical detachment and emotional engagement, thus enriching the understanding of professionals’ reactions to patient narratives. Additionally, the detailed analysis of a single patient experience film enabled in-depth examination and nuanced understanding, providing a solid foundation for future research.

Limitations of this study must also be considered. This study took place in the Netherlands, where it is culturally accepted to discuss mental health topics. The reliance on a single film may restrict the generalizability of findings across diverse patient experiences. The sample, predominantly from hospital settings and from personal networks (of the researcher and the film creators), may not fully represent other psychiatric care contexts. Despite efforts to ensure analytical rigor through collaborative transcript reviews and reflective discussions, potential researcher bias stemming from personal connections remains a consideration.^23^ Steps were taken to mitigate these biases through transparency and ongoing reflexivity, including regular consultations with supervisors and collaborative analyses. Besides, the inter-coder reliability was minimal in this study.

Future research directions include replicating this study across various medical specialties and healthcare environments to assess broader applicability. Comparative studies could explore different patient experiences depicted in films, potentially identifying conditions where films are particularly impactful. Investigating educational designs that integrate moderated interactive elements, or structured discussions could further optimize films’ effectiveness as training tools. Addressing these research avenues could significantly enhance our understanding and use of patient experience films in healthcare professional education.

## Conclusion

Patient-experience films offer valuable educational potential, particularly for early-career mental health professionals. However, effective integration requires addressing systemic resistance through structural adjustments and targeted educational strategies.

## Data Availability

The data is stored in the Vrije Universiteit Amsterdam data repository. In line with ethical and confidentiality requirements, these materials are available from the corresponding author on reasonable request and subject to approval by the data custodian.*

## Appendix

### Interview topic focus group

#### Introduction

a. First, I would like to thank you all for participating in this study. In this session, you will watch a film titled “Zielsverduistering” by Sem de Boer, which portrays her experience with an eating disorder. The film has a duration of approximately 45 min. Following the screening, we will conduct a focus group discussion lasting about 45–60 min, during which we will explore various aspects of the film.
Please note that the audio of the focus group will be recorded and transcribed for research purposes. All participants will remain anonymous, and the recordings will be deleted after the study concludes. You have the right to withdraw from the focus group at any time and are not obligated to answer any questions that make you uncomfortable. If you agree to these terms, please sign the informed consent form, which reiterates this information. Once the forms are signed, we can begin.
b. Introduction Round: Could you please introduce yourself? Include your role and the length of time you have been working in mental health care.
c. Background: During your education, were patient experience films or other methods showcasing patient experiences utilized?
d. Have you previously watched patient experience films? If so, which ones and for what reasons?
e. We will now watch the film. If you have any questions about Sem or the film’s content regarding her experience, please write them down. You can email your questions directly to Sem at her@email.

#### Reactions to the film

a. What are your initial reactions after watching the film?
b. Can you describe how you felt during the viewing? Did you experience any physical sensations?
c. Were there specific scenes or moments that impacted you more or less? Please elaborate.
d. Which challenges depicted in the film align with your professional experiences?
e. Were there parts of the film where you felt the patient’s experience during a mental health crisis was oversimplified or exaggerated?

#### Impact of the film

Please respond to the following statements using a scale from “Strongly Disagree” to “Strongly Agree”:

a. The film made me aware of aspects of mental health care that I had previously overlooked.
b. As a healthcare provider, I feel compelled to initiate changes in the current system.
c. The film added little to the knowledge I already had about mental health crises.
d. The portrayal of family and close relations in the film did not match my practical experiences.

#### Film as an educational tool

a. Do you think watching patient experience films can serve as a form of continuing education?
b. Would you recommend using this or similar films in the training of healthcare professionals?
c. What are the limitations of using films created from the patient’s perspective as educational tools for healthcare providers?

#### Conclusion

a. What would you like to convey to “continuing education programs for colleagues” and what message would you like to share with Sem?
b. Are there any final thoughts you wish to share?
c. Thank you once again for your participation.

### Interview topic list solo interview

#### Introduction

a. Thank you for participating in this study. This interview will be audio-recorded and transcribed. Your identity will remain anonymous, and the recordings will be deleted after the study concludes. You may withdraw from the interview at any time and are not obligated to answer any questions that make you uncomfortable. If you agree to these terms, please sign the informed consent form, which reiterates this information. Once the form is signed, we can begin.
b. Introduction Round: Could you please introduce yourself? Include your role and the length of time you have been working in mental health care.
c. Background: During your education, were patient experience films or other methods showcasing patient experiences utilized?
d. Have you previously watched patient experience films?

#### Reactions to the film

a. Can you describe how you felt during the viewing? Did you experience any physical sensations?
b. After several months, are there any feelings that have persisted?
c. Were there specific scenes or moments that impacted you more or less?
d. Which challenges depicted in the film align with your professional experiences?
e. Were there parts of the film where you felt the patient’s experience during a mental health crisis was oversimplified or exaggerated?

#### Impact of the film

Please respond to the following statements using a scale from “Strongly Disagree” to “Strongly Agree”:

a. The film made me aware of aspects of mental health care that I had previously overlooked.
b. As a healthcare provider, I feel compelled to initiate changes in the current system.
c. The film added little to the knowledge I already had about mental health crises.
d. The portrayal of family and close relations in the film did not match my practical experiences.

#### Film as an educational tool

a. Do you think watching patient experience films can serve as a form of continuing education?
b. Would you recommend using this or similar films in the training of healthcare professionals?
c. What are the limitations of using films created from the patient’s perspective as educational tools for healthcare providers?

#### Conclusion

a. Are there any final thoughts you wish to share?
b. Thank you once again for your participation.
